# Dietary Inflammatory Index, cardiovascular health, and long-term outcomes in hypertension: external validation in a Chinese percutaneous coronary intervention cohort

**DOI:** 10.3389/fnut.2026.1880961

**Published:** 2026-07-28

**Authors:** Jianchun Zhang, Yuwei Qiang, Ping Li, Qingqing Wu, Jiahui Song, Lu Yang

**Affiliations:** Department of Cardiology, Beijing Luhe Hospital, Capital Medical University, Beijing, China

**Keywords:** cardiovascular health, Dietary Inflammatory Index, hypertension, inflammation, life’s essential, percutaneous coronary intervention

## Abstract

**Background:**

Hypertension frequently coexists with poor cardiovascular health (CVH), yet whether the inflammatory potential of habitual diet, captured by the Dietary Inflammatory Index (DII), relates to CVH and to subsequent adverse cardiovascular events in this population remains unclear.

**Methods:**

A discovery analysis was performed in 12,376 hypertensive adults from NHANES (2005–2018), with suboptimal CVH defined as a Life’s Essential 8 (LE8) score < 80. External validation used a single-center retrospective cohort of 1,053 Chinese hypertensive patients who underwent percutaneous coronary intervention (PCI); because an LE8 ≥ 80 is rarely attained in this high-risk secondary-prevention population, suboptimal CVH was defined as LE8 < 60. Multivariable logistic regression, restricted cubic spline, subgroup, and mediation analyses examined the DII–CVH association in both cohorts; NHANES analyses incorporated complex survey design weights. In the PCI cohort, Cox proportional-hazards models assessed the associations of DII and LE8 with major adverse cardiovascular events (MACE), all-cause mortality, recurrent myocardial infarction (MI), and repeat revascularization.

**Results:**

In NHANES, higher DII was independently associated with suboptimal CVH [fully adjusted odds ratio (OR) 1.39, 95% confidence interval (CI) 1.30–1.50; OR 3.84, 95% CI 2.67–5.52 for the highest versus lowest DII tertile], with a linear dose–response. A consistent association was observed in the PCI cohort: each one-unit increase in DII conferred 29% higher odds of suboptimal CVH (fully adjusted OR 1.29, 95% CI 1.19–1.39; OR 2.99, 95% CI 2.11–4.22 for the highest versus lowest tertile), with a linear dose–response. DII independently predicted MACE [hazard ratio (HR) 1.19, 95% CI 1.10–1.28], recurrent MI (HR 1.24, 95% CI 1.10–1.39), repeat revascularization (HR 1.14, 95% CI 1.02–1.28), and all-cause mortality (HR 1.19, 95% CI 1.04–1.35).

**Conclusion:**

Higher dietary inflammatory potential is linearly associated with poorer CVH across two demographically and clinically distinct hypertensive cohorts and predicts adverse cardiovascular events in patients with established coronary disease, warranting prospective evaluation.

## 1 Introduction

Hypertension is a leading contributor to the global cardiovascular disease (CVD) burden and a primary determinant of heart failure, coronary artery disease (CAD), atrial fibrillation, valvular disorders, and stroke (1, 2). In 2022, the American Heart Association (AHA) introduced Life’s Essential 8 (LE8) as a more granular framework for cardiovascular health (CVH), integrating four health behaviors (diet, physical activity, nicotine exposure, sleep) and four health factors [body-mass index (BMI)], blood lipids, blood glucose, blood pressure] (3). Higher LE8 scores have been linked to lower cardiovascular event rates and mortality, including recent prospective cohort evidence in hypertensive populations (4, 5); given the low prevalence of ideal CVH globally (6), strategies that promote it are central to improving long-term prognosis.

Dietary patterns are major determinants of CVH and a tractable target for intervention. The Mediterranean diet, characterized by whole grains, fruits, vegetables, and fish, reduces cardiovascular risk and metabolic dysfunction (7, 8) and is associated with lower platelet and leukocyte counts (9), implicating diet-induced low-grade inflammation as a candidate mechanism. To quantify the inflammatory potential of habitual intake, Shivappa and colleagues developed the Dietary Inflammatory Index (DII), which integrates 28 food parameters and shows robust associations with circulating inflammatory markers including C-reactive protein, interleukin-6, and homocysteine (10–12). Mechanistically, these inflammatory mediators promote endothelial activation, vascular inflammation, and oxidative stress, the processes that initiate and destabilize atherosclerotic lesions, and they also contribute to insulin resistance, dyslipidaemia, and adiposity (13). A pro-inflammatory diet may therefore influence cardiovascular health beyond its dietary domain alone, plausibly extending to the metabolic and vascular factors, namely blood glucose, blood lipids, body-mass index, and blood pressure, that are captured within the LE8 score.

Inflammation is a central mechanism in hypertensive vascular injury (14); as hypertension is itself a state of chronic vascular inflammation, hypertensive patients may be particularly susceptible to a pro-inflammatory diet. A higher DII has been linked to the prevalence of hypertension (15) and to chronic kidney disease, sarcopenia, and elevated mortality among hypertensive individuals (16–18), whereas Mediterranean-style dietary patterns reduce blood pressure (19). Beyond hypertension, a pro-inflammatory diet has been associated with cardiovascular disease incidence and mortality in general populations (20) and with all-cause mortality among adults with coronary heart disease (21); however, the latter evidence derives largely from survey cohorts with self-reported disease and all-cause death. Two questions therefore remain. First, whether the DII relates to overall CVH, as captured by the composite LE8 score, in hypertension, and whether this relationship generalizes to an independent, ethnically distinct, and clinically higher-risk cohort, has not been established. Second, whether the DII predicts hard cardiovascular events, including major adverse cardiovascular events, recurrent myocardial infarction, and repeat revascularization, in hypertensive patients with angiographically confirmed coronary disease, remains unknown.

We therefore conducted a two-stage analysis. The discovery analysis used data from 12,376 hypertensive adults in the National Health and Nutrition Examination Survey (NHANES) 2005–2018 to characterize the DII–CVH association and its candidate mediators. External validation was then performed in an independent, single-center retrospective cohort of 1,053 Chinese hypertensive patients who underwent percutaneous coronary intervention (PCI); in this cohort, the analysis was extended to adverse cardiovascular endpoints, including major adverse cardiovascular events (MACE), all-cause mortality, recurrent myocardial infarction (MI), and repeat revascularization.

## 2 Materials and methods

### 2.1 Discovery cohort: NHANES population

The discovery analysis used data from NHANES, a continuous, multistage, stratified, probability-based survey conducted by the U.S. National Center for Health Statistics (NCHS) and the Centers for Disease Control and Prevention (CDC) to assess the health and nutritional status of the non-institutionalized U.S. population. The protocol was approved by the NCHS Research Ethics Review Board, and all participants provided written informed consent.

**FIGURE 1:**
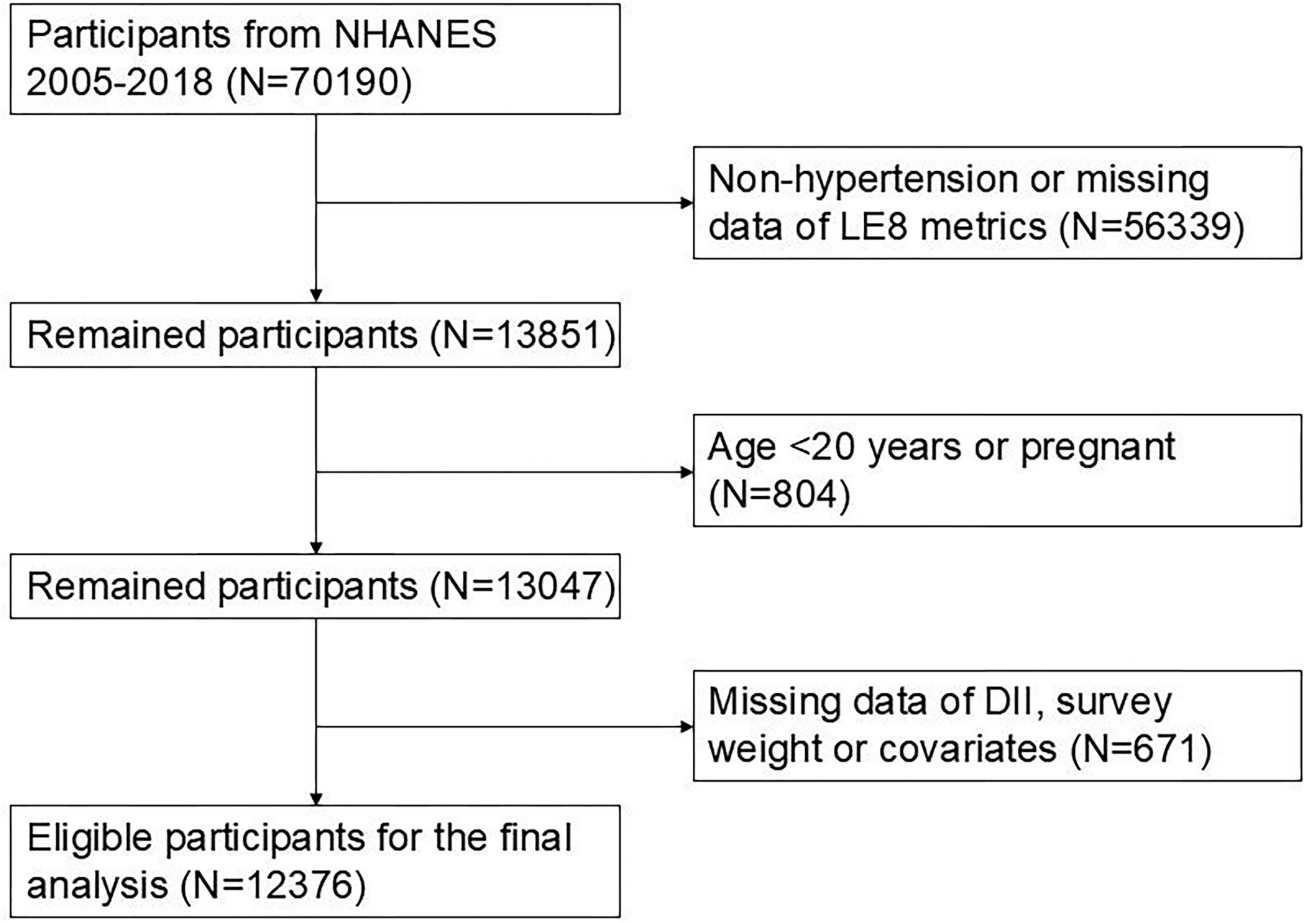
Study flow diagram. The discovery analysis included 12,376 hypertensive adults from NHANES cycles 2005–2018 with complete data on cardiovascular health metrics and the Dietary Inflammatory Index. External validation was performed in 1,053 Chinese hypertensive patients who underwent percutaneous coronary intervention at Beijing Luhe Hospital, with retrospective ascertainment of adverse cardiovascular outcomes.

Adults with hypertension from the 2005 to 2018 NHANES cycles with complete data on CVH metrics were included. Of 13,851 participants initially identified, 804 were excluded because of age < 20 years or pregnancy, and 671 were excluded for missing data on DII components, survey weights, or relevant covariates, leaving 12,376 participants for analysis (Figure 1).

### 2.2 External validation cohort: Chinese hypertensive PCI population

The validation cohort comprised 1,053 consecutive Chinese adults with hypertension who underwent PCI at Beijing Luhe Hospital (Capital Medical University) between June 2020 and June 2023. Adverse cardiovascular outcomes were ascertained retrospectively through systematic review of electronic medical records, the institutional cardiovascular registry, and structured telephone follow-up. Eligibility required (i) hypertension diagnosed before the index PCI in accordance with the 2024 European Society of Cardiology (ESC) guidelines for the management of elevated blood pressure and hypertension (22), defined as one or more of the following: a documented prior physician diagnosis of hypertension recorded in the electronic medical record, current use of antihypertensive medication, or repeated office blood pressure measurements meeting the diagnostic threshold of systolic blood pressure ≥ 140 mmHg or diastolic blood pressure ≥ 90 mmHg, which is concordant with the NHANES diagnostic criteria and ensures direct cross-cohort comparability; (ii) successful PCI with detailed clinical, biochemical, and dietary data available; and (iii) age ≥ 20 years. Patients with active malignancy or severe hepatic or renal failure were excluded. The study was approved by the Institutional Review Board of Beijing Luhe Hospital, which granted a waiver of informed consent given the retrospective design, the use of de-identified data, and the minimal risk to participants. The study was conducted in accordance with the Declaration of Helsinki. Variables, definitions, and statistical models were harmonized with NHANES wherever possible to enable direct cross-cohort comparison.

### 2.3 Definition of hypertension and cardiovascular health

In NHANES, hypertension was identified when one or more of the following were met: (1) self-reported physician diagnosis of hypertension; (2) mean systolic blood pressure ≥ 140 mmHg; (3) mean diastolic blood pressure ≥ 90 mmHg; or (4) current use of antihypertensive medication (23). Blood pressure was measured by trained personnel using standardized procedures. In the validation cohort, hypertension was defined according to the 2024 ESC guidelines as a documented prior physician diagnosis of hypertension, current use of antihypertensive medication, or repeated office blood pressure measurements meeting the diagnostic threshold of systolic blood pressure ≥ 140 mmHg or diastolic blood pressure ≥ 90 mmHg (22). Both definitions share the same office-BP threshold ( ≥ 140/90 mmHg), ensuring direct cross-cohort comparability.

CVH was quantified using the AHA LE8 score, which integrates four health behaviors (diet, physical activity, nicotine exposure, sleep) and four health factors (BMI, blood lipids, blood glucose, blood pressure) (3). The Healthy Eating Index–2015 (HEI-2015) was used to score diet quality (24); the remaining seven components were derived as follows: physical activity from self-reported moderate-to-vigorous physical activity, nicotine exposure from self-reported tobacco use and second-hand smoke exposure, sleep from self-reported habitual sleep duration, BMI from measured height and weight, blood lipids from non-high-density-lipoprotein cholesterol, blood glucose from fasting plasma glucose or glycated hemoglobin together with diabetes status, and blood pressure from measured systolic and diastolic values together with antihypertensive use. Each component was scored 0–100 according to the AHA algorithm, and the LE8 composite was computed as the unweighted mean of the eight components, also on a 0–100 scale.

In NHANES, suboptimal CVH was defined as LE8 < 80, in line with the AHA classification of high CVH ( ≥ 80) and prior literature (3, 25). In the PCI cohort, all patients had established coronary disease and an LE8 ≥ 80 was rare; suboptimal CVH was therefore prespecified as LE8 < 60 to permit meaningful within-cohort stratification, an approach analogous to that used in other secondary-prevention populations (26, 27). An alternative LE8 < 50 threshold was tested in NHANES sensitivity analyses to verify the robustness of the discovery findings (Supplementary Table 1).

### 2.4 Calculation of the Dietary Inflammatory Index

In NHANES, dietary intake was assessed by two non-consecutive 24-h dietary recalls administered by trained interviewers. The DII was computed using 28 food parameters covering energy, macronutrients (carbohydrate, protein, fats), vitamins (A, B-complex, C, D, E), minerals (iron, magnesium, zinc, selenium), fiber, caffeine, and other dietary components, in accordance with the algorithm of Shivappa et al. (10, 28). Briefly, each parameter was Z-standardized against published global reference means and standard deviations, converted to a centered percentile, multiplied by the literature-derived inflammatory effect score, and summed across all parameters to yield a single DII value, with higher scores indicating a more pro-inflammatory diet.

In the validation cohort, dietary intake was assessed by trained interviewers at the bedside during the index admission using two consecutive 24-h dietary recalls covering the 48 h immediately preceding hospitalization (i.e., the day before admission and the day before that). This baseline-at-admission approach has been adopted in similar cohorts of patients with established coronary disease (29). Reported foods and portions were converted into nutrient intakes using the China Food Composition Tables, 6th Standard Edition (30), and the resulting nutrient values were then mapped onto the same 28 food parameters defined by Shivappa et al. (10). The DII was computed using the identical algorithm and the same Shivappa global reference means and standard deviations as in NHANES (10) to ensure cross-cohort methodological comparability.

### 2.5 Outcomes for prognostic analysis

In the PCI validation cohort, patients were followed from the index PCI, and prespecified outcomes were ascertained through systematic review of electronic medical records, the institutional cardiovascular registry, and structured telephone follow-up. Outcomes were independently adjudicated by two cardiologists blinded to DII status. The primary outcome was MACE, defined as a composite of all-cause death, recurrent MI, and repeat revascularization. Secondary outcomes were the individual components of MACE. Recurrent MI was diagnosed in accordance with the Fourth Universal Definition of Myocardial Infarction. Repeat revascularization was defined as any clinically driven percutaneous or surgical coronary intervention occurring after the index PCI. Time to event was measured from the index PCI to the first occurrence of the relevant endpoint, and patients without an event were censored at the date of last contact; the median follow-up was 23.9 months (interquartile range 19.0–29.5).

### 2.6 Statistical analyses

All analyses were conducted on complete cases: in both cohorts, participants were included only if they had complete data on the DII, the LE8 components, and the covariates required for the relevant models, and missing data were not imputed. Categorical variables are presented as frequencies (percentages). Continuous variables are summarized as mean ± standard deviation in the PCI cohort and as survey-weighted mean ± standard error in NHANES, where the complex survey design weights of NHANES were applied throughout; continuous variables with a skewed distribution are summarized as median [interquartile range]. Between-group comparisons across DII tertiles used one-way ANOVA for continuous variables (or the Kruskal–Wallis test for skewed variables) and Pearson χ^2^ test for categorical variables in the PCI cohort, and the corresponding design-based tests (Wald F-test and survey-weighted χ^2^ test) accounting for NHANES sampling weights and complex survey design.

Participants were classified into DII tertiles within each cohort. The association between DII and suboptimal CVH was estimated by logistic regression using three prespecified models. NHANES analyses (including all logistic regression, restricted cubic spline [RCS], subgroup, and mediation analyses) incorporated NHANES sampling weights and the complex survey design throughout. Because the DII was derived from two 24-h dietary recalls, the 2-day dietary sample weight (WTDR2D) was applied, rescaled by a factor of 1/7 to combine the seven 2-year cycles spanning 2005–2018; the masked variance pseudo-stratum (SDMVSTRA) and pseudo-primary sampling unit (SDMVPSU) were specified in the survey design object, with variances estimated by Taylor-series linearization. Analyses in the PCI cohort, which is a single-center clinical cohort not drawn from a complex survey, were conducted on unweighted data. In NHANES, Model 1 was unadjusted; Model 2 additionally adjusted for age, sex, race, education, BMI, smoking status, and poverty income ratio (PIR); Model 3 further added diabetes, CAD, and stroke. In the PCI cohort, Model 1 was unadjusted; Model 2 adjusted for age and sex; Model 3 (fully adjusted) additionally accounted for current smoking, diabetes mellitus, prior MI, prior stroke, and estimated glomerular filtration rate (eGFR). Covariate selection was tailored to the distinct clinical settings of the two populations.

Restricted cubic spline (RCS) analyses with four knots placed at the 5th, 35th, 65th, and 95th percentiles of DII were used to characterize dose–response relationships, with the reference set at the cohort-specific median DII; tests for non-linearity used the Wald χ^2^ statistic for the cubic terms. Subgroup analyses examined effect modification by sex, age, race, education, PIR, BMI, smoking, diabetes, and, where applicable, admission diagnosis [ST-elevation myocardial infarction (STEMI)/non-ST-elevation myocardial infarction (NSTEMI) vs. unstable angina] and prior MI; multiplicative DII × subgroup interaction terms in the fully adjusted models were used to test for formal interaction. Because these interactions were tested across multiple strata, the subgroup analyses were regarded as exploratory. Mediation analysis was performed with the R package “mediation,” with neutrophil count, white blood cell (WBC) count, and BMI prespecified as candidate mediators in both cohorts. Indirect effects were estimated with the non-parametric bootstrap (500 resamples) and percentile-based 95% confidence intervals.

In the PCI cohort, Cox proportional-hazards models estimated the prognostic associations of DII (per one-unit increase) and LE8 (per 10-unit increase) with MACE, all-cause mortality, recurrent MI, and repeat revascularization, adjusted for age, sex, current smoking, diabetes mellitus, prior MI, prior stroke, eGFR, and the Synergy between Percutaneous Coronary Intervention with Taxus and Cardiac Surgery (SYNTAX) score. The proportional-hazards assumption was verified using Schoenfeld residuals. Cumulative event-free survival across DII tertiles was visualized using Kaplan–Meier curves with log-rank testing.

Sensitivity analyses in NHANES redefined suboptimal CVH as LE8 < 50 (Supplementary Table 1). All analyses were performed in Stata 15.0 (StataCorp, College Station, TX, United States) and R version 4.3.1 (R Foundation for Statistical Computing, Vienna, Austria). Two-sided *P* < 0.05 was considered statistically significant.

## 3 Results

### 3.1 Discovery cohort: NHANES baseline characteristics

Baseline characteristics of the 12,376 NHANES hypertensive participants are presented in Table 1. The weighted mean age was 56.9 years and 48.0% were men. Compared with participants in the lowest DII tertile, those in the highest tertile were more often women, more likely to have diabetes or stroke, and had higher BMI, WBC count, and neutrophil count, and lower PIR and LE8 scores. The HEI-2015, physical-activity, nicotine-exposure, and sleep-health subscores all decreased monotonically across DII tertiles, consistent with broader lifestyle clustering with diet quality.

**TABLE 1:** Baseline characteristics of the NHANES discovery cohort, stratified by tertiles of the Dietary Inflammatory Index (*n* = 12,376; weighted).

Characteristic	Total (*n* = 12,376)	T1 (*n* = 3,795)	T2 (*n* = 4,027)	T3 (*n* = 4,554)	*P*
Age, years	56.86 ± 0.28	56.46 ± 0.45	56.99 ± 0.39	57.13 ± 0.40	0.432
Male sex, n (%)	5,939 (48.0)	2,356 (62.1)	1,957 (48.6)	1,626 (35.7)	< 0.001
Race/ethnicity, n (%)					< 0.001
Mexican American	714 (5.8)	247 (6.5)	226 (5.6)	241 (5.3)	
Other Hispanic	494 (4.0)	159 (4.2)	157 (3.9)	178 (3.9)	
Non-Hispanic White	8,687 (70.2)	2,736 (72.1)	2,895 (71.9)	3,056 (67.1)	
Non-Hispanic Black	1,668 (13.5)	376 (9.9)	495 (12.3)	797 (17.5)	
Other	813 (6.6)	277 (7.3)	254 (6.3)	282 (6.2)	
Education, n (%)					< 0.001
Less than high school	5,333 (43.1)	1,271 (33.5)	1,707 (42.4)	2,355 (51.7)	
High school	3,931 (31.8)	1,195 (31.5)	1,297 (32.2)	1,439 (31.6)	
Above high school	3,112 (25.1)	1,329 (35.0)	1,023 (25.4)	760 (16.7)	
Poverty income ratio	2.97 ± 0.04	3.31 ± 0.05	3.00 ± 0.05	2.61 ± 0.05	< 0.001
BMI category, n (%)					< 0.001
≤ 25 kg/m^2^	2,394 (19.3)	804 (21.2)	693 (17.2)	897 (19.7)	
25–30 kg/m^2^	3,898 (31.5)	1,249 (32.9)	1,369 (34.0)	1,280 (28.1)	
> 30 kg/m^2^	6,084 (49.2)	1,742 (45.9)	1,965 (48.8)	2,377 (52.2)	
Current smoking, n (%)	6,142 (49.6)	1,844 (48.6)	2,021 (50.2)	2,277 (50.0)	0.589
Diabetes, n (%)	2,748 (22.2)	766 (20.2)	894 (22.2)	1,088 (23.9)	0.018
Coronary heart disease, n (%)	867 (7.0)	262 (6.9)	286 (7.1)	319 (7.0)	0.328
Stroke, n (%)	714 (5.8)	152 (4.0)	221 (5.5)	341 (7.5)	< 0.001
WBC count, × 10^9^/L	7.51 ± 0.05	7.35 ± 0.07	7.51 ± 0.06	7.67 ± 0.08	0.014
Neutrophil count, × 10^9^/L	4.47 ± 0.03	4.36 ± 0.04	4.50 ± 0.05	4.56 ± 0.05	0.005
HEI-2015 score	40.19 ± 0.60	57.22 ± 0.84	38.93 ± 0.76	23.65 ± 0.63	< 0.001
Physical activity score	64.66 ± 0.69	69.97 ± 1.12	64.55 ± 1.19	59.20 ± 1.12	< 0.001
Nicotine exposure score	71.16 ± 0.64	74.71 ± 1.08	70.75 ± 0.96	67.88 ± 1.14	< 0.001
Sleep health score	81.23 ± 0.40	84.28 ± 0.57	80.87 ± 0.64	78.41 ± 0.74	< 0.001
BMI score	50.76 ± 0.58	52.88 ± 1.02	50.11 ± 0.97	49.19 ± 0.83	0.005
Blood lipid score	58.33 ± 0.51	59.59 ± 1.00	57.90 ± 0.76	57.45 ± 0.80	0.164
Blood glucose score	76.39 ± 0.42	78.19 ± 0.63	75.87 ± 0.73	75.05 ± 0.74	0.001
Blood pressure score	44.54 ± 0.48	45.01 ± 0.86	43.38 ± 0.81	45.22 ± 0.69	0.169
DII score	1.45 ± 0.04	−0.69 ± 0.03	1.70 ± 0.01	3.45 ± 0.02	< 0.001
LE8 score	60.91 ± 0.23	65.11 ± 0.34	60.29 ± 0.36	57.01 ± 0.39	< 0.001

Continuous variables are weighted mean ± standard error; categorical variables are n (weighted %). P values are derived from design-based Wald *F*-tests for continuous variables and survey-weighted χ^2^-tests for categorical variables. BMI, body-mass index; DII, Dietary Inflammatory Index; HEI-2015, Healthy Eating Index 2015; LE8, Life’s Essential 8; T1/T2/T3, DII tertile 1/2/3; WBC, white blood cell.

### 3.2 Discovery cohort: DII and suboptimal CVH

**FIGURE 2:**
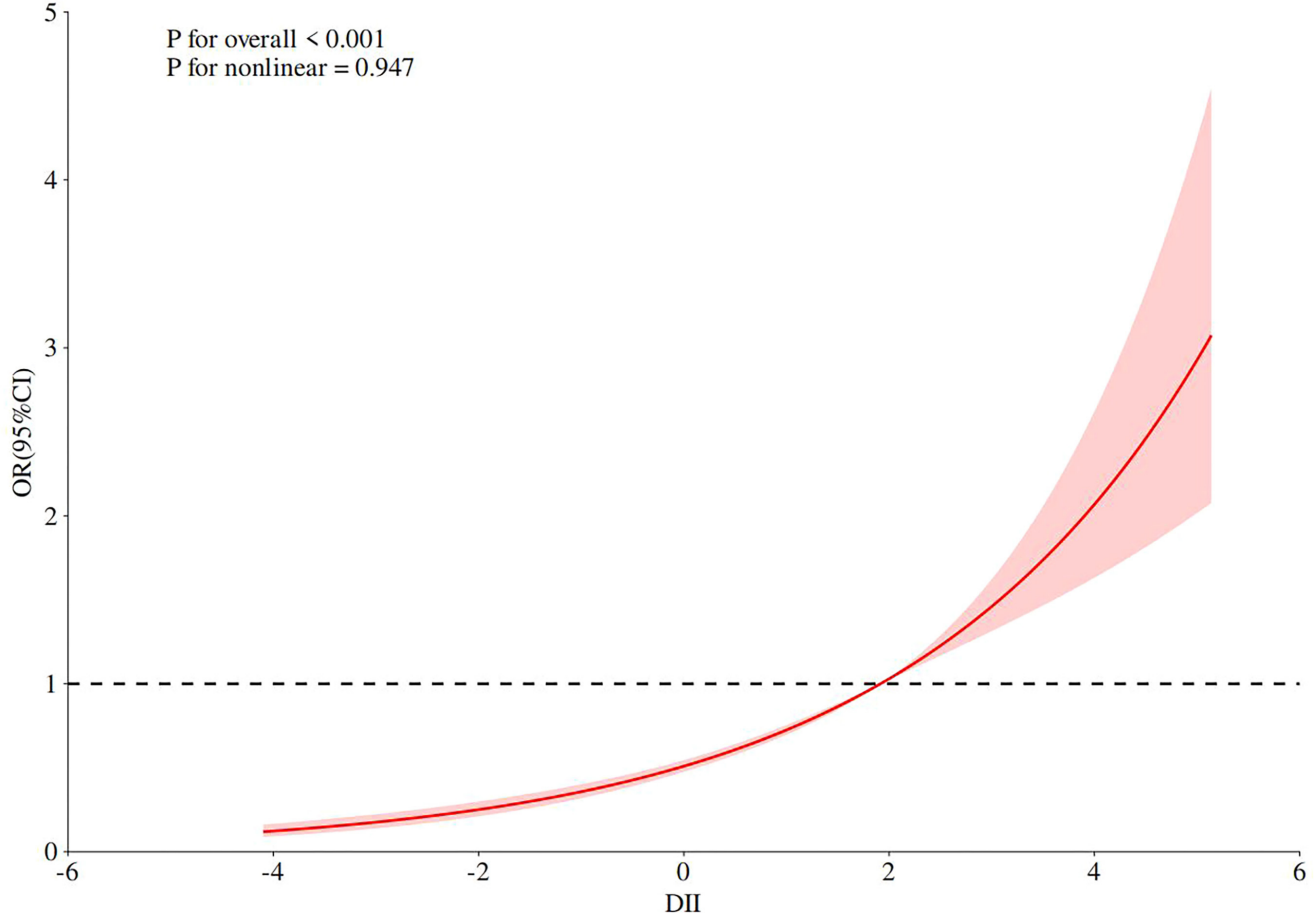
Restricted cubic spline analysis of the dose–response association between the Dietary Inflammatory Index (DII) and the odds of suboptimal cardiovascular health (LE8 < 80) in the NHANES discovery cohort (n = 12,376). The model was adjusted for age, sex, race, education, body-mass index, smoking status, poverty income ratio, diabetes, coronary artery disease, and stroke, and incorporated NHANES sampling weights. Four knots were placed at the 5th, 35th, 65th, and 95th percentiles of DII, with the reference value (OR = 1.00) set at the median DII. The solid line denotes the adjusted odds ratio and the shaded area the 95% confidence interval. P for non-linearity = 0.947.

Higher DII was independently associated with suboptimal CVH (Table 2, NHANES panel). In the unadjusted model, each one-unit increase in DII conferred 46% higher odds of suboptimal CVH (OR 1.46, 95% CI 1.36–1.55); the association persisted after multivariable adjustment (Model 3 OR 1.39, 95% CI 1.30–1.50). When DII was modeled categorically, participants in T2 and T3 had 2.54-fold (95% CI 1.92–3.36) and 3.84-fold (95% CI 2.67–5.52) higher odds of suboptimal CVH, respectively, compared with T1 (P for trend < 0.001). RCS analysis demonstrated a positive, monotonic dose–response relationship with no evidence of non-linearity (P for non-linear = 0.947; Figure 2).

**TABLE 2:** Multivariable logistic regression of the association between the dietary inflammatory index and suboptimal cardiovascular health in the NHANES discovery cohort and the Chinese PCI validation cohort.

Exposure	Model 1, OR (95% CI), P	Model 2, OR (95% CI), P	Model 3, OR (95% CI), P
NHANES discovery cohort (n = 12,376; suboptimal CVH defined as LE8 < 80)			
DII (per 1-unit)	1.46 (1.36, 1.55), < 0.001	1.40 (1.30, 1.50), < 0.001	1.39 (1.30, 1.50), < 0.001
DII tertiles			
T1 (lowest)	1.00 (Reference)	1.00 (Reference)	1.00 (Reference)
T2	2.63 (2.01, 3.43), < 0.001	2.49 (1.88, 3.29), < 0.001	2.54 (1.92, 3.36), < 0.001
T3 (highest)	4.10 (2.92, 5.74), < 0.001	3.89 (2.69, 5.61), < 0.001	3.84 (2.67, 5.52), < 0.001
P for trend	< 0.001	<0.001	< 0.001
Chinese PCI validation cohort (n = 1,053; suboptimal CVH defined as LE8 < 60)			
DII (per 1-unit)	1.22 (1.14, 1.31), < 0.001	1.24 (1.15, 1.33), < 0.001	1.29 (1.19, 1.39), < 0.001
DII tertiles			
T1 (lowest)	1.00 (Reference)	1.00 (Reference)	1.00 (Reference)
T2	1.42 (1.05, 1.91), 0.021	1.49 (1.11, 2.02), 0.009	1.67 (1.20, 2.33), 0.002
T3 (highest)	2.40 (1.76, 3.27), < 0.001	2.51 (1.83, 3.44), < 0.001	2.99 (2.11, 4.22), < 0.001
P for trend	< 0.001	<0.001	< 0.001

NHANES analyses incorporated complex survey-design weights. Model 1: unadjusted. Model 2 (NHANES): age, sex, race, education, BMI, smoking status, and PIR; Model 2 (PCI): age and sex. Model 3 (NHANES): Model 2 covariates plus diabetes, CAD, and stroke; Model 3 (PCI): Model 2 covariates plus current smoking, diabetes mellitus, prior MI, prior stroke, and eGFR. P for trend was estimated by entering DII tertiles as an ordinal variable (1, 2, 3). CAD, coronary artery disease; CI, confidence interval; CVH, cardiovascular health; DII, Dietary Inflammatory Index; eGFR, estimated glomerular filtration rate; LE8, Life’s Essential 8; MI, myocardial infarction; OR, odds ratio; PCI, percutaneous coronary intervention; PIR, poverty income ratio; T1/T2/T3, DII tertile 1/2/3.

### 3.3 Discovery cohort: subgroup, mediation, and sensitivity analyses

Subgroup analyses were prespecified as exploratory. Nominally significant interactions were observed in the smoking and diabetes strata (Pinteraction < 0.05), with stronger DII–CVH associations among smokers and non-diabetic participants (Supplementary Figure 1); given the number of strata tested, these interactions are interpreted as hypothesis-generating rather than confirmatory. Mediation analysis showed that neutrophil count and BMI partially mediated the DII–CVH association, with proportions mediated of 2.02% (*P* < 0.001) and 7.83% (*P* < 0.001), respectively (Supplementary Table 2); the direct effect of DII on CVH remained dominant. The associations between DII and each candidate mediator, and between each mediator and suboptimal CVH, are detailed in Supplementary Tables 3, 4, respectively. Sensitivity analyses redefining suboptimal CVH as LE8 < 50 yielded consistent results (Supplementary Table 1).

### 3.4 External validation cohort: baseline characteristics

Baseline characteristics of the 1,053 PCI participants are presented in Table 3. Compared with patients in T1, those in T3 were younger (60.2 ± 11.2 vs. 63.1 ± 10.6 years; *P* = 0.002) and had higher total cholesterol, low-density lipoprotein (LDL) cholesterol, WBC count, neutrophil count, and high-sensitivity C-reactive protein (hsCRP) (all *P* ≤ 0.011). Demographic characteristics, blood pressure, glycaemic indices, eGFR, comorbidities, admission diagnosis, SYNTAX score, and baseline medication use were comparable across tertiles (all *P* > 0.05). The LE8 score decreased progressively from T1 to T3 (*P* < 0.001).

**TABLE 3:** Baseline characteristics of the Chinese percutaneous coronary intervention validation cohort, stratified by tertiles of the Dietary Inflammatory Index (*n* = 1,053).

Characteristic	Overall (*n* = 1,053)	T1 (*n* = 359)	T2 (*n* = 347)	T3 (*n* = 347)	*P*
Age, years	61.50 ± 11.02	63.08 ± 10.59	61.14 ± 11.14	60.23 ± 11.18	0.002
Sex, n (%)					0.088
Male	836 (79.4)	274 (76.3)	288 (83.0)	274 (79.0)	
Female	217 (20.6)	85 (23.7)	59 (17.0)	73 (21.0)	
Body-mass index, kg/m^2^	26.03 ± 3.79	25.71 ± 3.80	26.21 ± 3.70	26.18 ± 3.85	0.166
Systolic blood pressure, mmHg	137.20 ± 14.03	137.82 ± 14.75	137.65 ± 13.29	136.11 ± 13.95	0.208
Diastolic blood pressure, mmHg	81.83 ± 10.28	81.57 ± 10.09	81.76 ± 10.13	82.15 ± 10.64	0.749
Total cholesterol, mmol/L	4.09 ± 1.13	4.02 ± 1.20	4.01 ± 1.09	4.26 ± 1.10	0.005
HDL cholesterol, mmol/L	1.02 ± 0.24	1.03 ± 0.24	1.00 ± 0.22	1.04 ± 0.26	0.119
LDL cholesterol, mmol/L	2.44 ± 0.93	2.35 ± 0.88	2.40 ± 0.96	2.56 ± 0.96	0.011
Triglycerides, mmol/L	1.79 ± 1.31	1.75 ± 1.43	1.70 ± 1.04	1.93 ± 1.40	0.058
HbA1c, %	6.81 ± 1.35	6.82 ± 1.42	6.80 ± 1.31	6.81 ± 1.32	0.994
Fasting glucose, mmol/L	7.23 ± 2.85	7.01 ± 2.85	7.29 ± 2.87	7.40 ± 2.83	0.178
WBC count, × 10^9^/L	7.77 ± 2.36	7.19 ± 1.96	7.92 ± 2.37	8.22 ± 2.61	< 0.001
Neutrophil count, × 10^9^/L	5.31 ± 2.14	4.79 ± 1.71	5.42 ± 2.15	5.73 ± 2.42	< 0.001
hsCRP, mg/L	2.37 [0.82–6.20]	1.64 [0.73–4.22]	2.38 [0.77–6.45]	3.15 [1.05–8.35]	< 0.001
eGFR, mL/min/1.73 m^2^	84.02 ± 23.68	82.07 ± 23.64	84.65 ± 22.59	85.40 ± 24.70	0.176
Diabetes mellitus, n (%)	451 (42.8)	159 (44.3)	151 (43.5)	141 (40.6)	0.588
Current smoker, n (%)	475 (45.1)	157 (43.7)	166 (47.8)	152 (43.8)	0.459
Prior MI, n (%)	240 (22.8)	90 (25.1)	75 (21.6)	75 (21.6)	0.448
Prior stroke, n (%)	107 (10.2)	29 (8.1)	36 (10.4)	42 (12.1)	0.206
STEMI on admission, n (%)	11 (1.0)	4 (1.1)	2 (0.6)	5 (1.4)	0.527
NSTEMI on admission, n (%)	105 (10.0)	41 (11.4)	40 (11.5)	24 (6.9)	0.068
SYNTAX score	22.15 ± 7.85	22.54 ± 7.92	22.24 ± 8.06	21.65 ± 7.57	0.313
Antihypertensive therapy, n (%)	884 (84.0)	299 (83.3)	292 (84.1)	293 (84.4)	0.910
Lipid-lowering therapy, n (%)	1,046 (99.3)	356 (99.2)	345 (99.4)	345 (99.4)	0.887
DII score	1.97 ± 1.82	0.02 ± 0.97	2.01 ± 0.43	3.96 ± 1.00	< 0.001
LE8 score	57.38 ± 9.32	59.78 ± 9.17	57.64 ± 8.41	54.64 ± 9.63	< 0.001

Continuous variables are mean ± standard deviation or median [interquartile range] for skewed distributions; categorical variables are n (%). *P*-values are derived from one-way ANOVA or the Kruskal–Wallis test, as appropriate, for continuous variables, and the Pearson χ^2^-test for categorical variables. DII, Dietary Inflammatory Index; eGFR, estimated glomerular filtration rate; HbA1c, glycated hemoglobin; HDL, high-density lipoprotein; hsCRP, high-sensitivity C-reactive protein; LDL, low-density lipoprotein; LE8, Life’s Essential 8; MI, myocardial infarction; NSTEMI, non-ST-elevation myocardial infarction; STEMI, ST-elevation myocardial infarction; SYNTAX, Synergy between Percutaneous Coronary Intervention with Taxus and Cardiac Surgery; T1/T2/T3, DII tertile 1/2/3; WBC, white blood cell.

### 3.5 External validation cohort: consistency of the DII–CVH association

**FIGURE 3:**
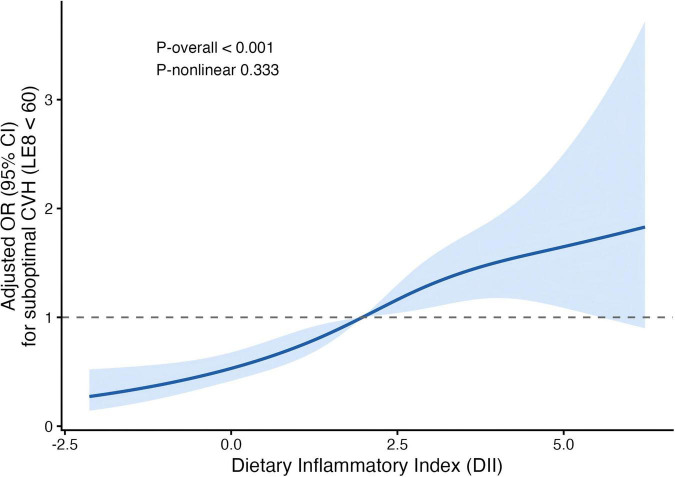
Restricted cubic spline analysis of the dose–response association between the Dietary Inflammatory Index (DII) and the odds of suboptimal cardiovascular health (LE8 < 60) in the Chinese PCI validation cohort (n = 1,053). The model was adjusted for age, sex, current smoking, diabetes mellitus, prior myocardial infarction, prior stroke, and estimated glomerular filtration rate. Four knots were placed at the 5th, 35th, 65th, and 95th percentiles of DII, with the reference value (OR = 1.00) set at the median DII. The solid line denotes the adjusted odds ratio and the shaded area the 95% confidence interval. P-overall < 0.001; P-non-linear = 0.333.

The DII–CVH association was directionally consistent in the PCI cohort (Table 2, PCI panel). Each one-unit increase in DII conferred 22% higher unadjusted odds of suboptimal CVH (OR 1.22, 95% CI 1.14–1.31), with the association strengthening after adjustment for age and sex (Model 2 OR 1.24, 95% CI 1.15–1.33) and reaching its maximum in the fully adjusted model (Model 3 OR 1.29, 95% CI 1.19–1.39). Categorical analyses showed a stepwise gradient: relative to T1, participants in T2 and T3 had 1.67-fold (95% CI 1.20–2.33) and 2.99-fold (95% CI 2.11–4.22) higher fully adjusted odds of suboptimal CVH, respectively (P for trend < 0.001). The RCS curve confirmed a linear, monotonic dose–response (P-overall < 0.001; P-non-linear = 0.333; Figure 3).

### 3.6 External validation cohort: subgroup and mediation analyses

In the PCI cohort, the DII–CVH association was directionally consistent across all prespecified strata, with point estimates ranging from OR 1.24 to 1.38 (Supplementary Table 5). The smoking and diabetes interactions observed in NHANES did not replicate (Pinteraction = 0.555 and 0.261, respectively).

Mediation analyses in the PCI cohort tested neutrophil count, BMI, and WBC count as candidate mediators (Supplementary Table 6). Indirect effects were directionally consistent with NHANES (neutrophil count: indirect effect 0.0018, 95% CI –0.0018 to 0.0056, proportion mediated 3.30%; BMI: indirect effect 0.0022, 95% CI –0.0024 to 0.0069, proportion mediated 4.05%; WBC count: indirect effect 0.0015, 95% CI –0.0018 to 0.0053, proportion mediated 2.80%), but none reached statistical significance (all P > 0.05). The direct effect of DII on CVH remained statistically significant in every mediation model (all *P* < 0.001).

### 3.7 Long-term outcomes in the PCI validation cohort

**FIGURE 4:**
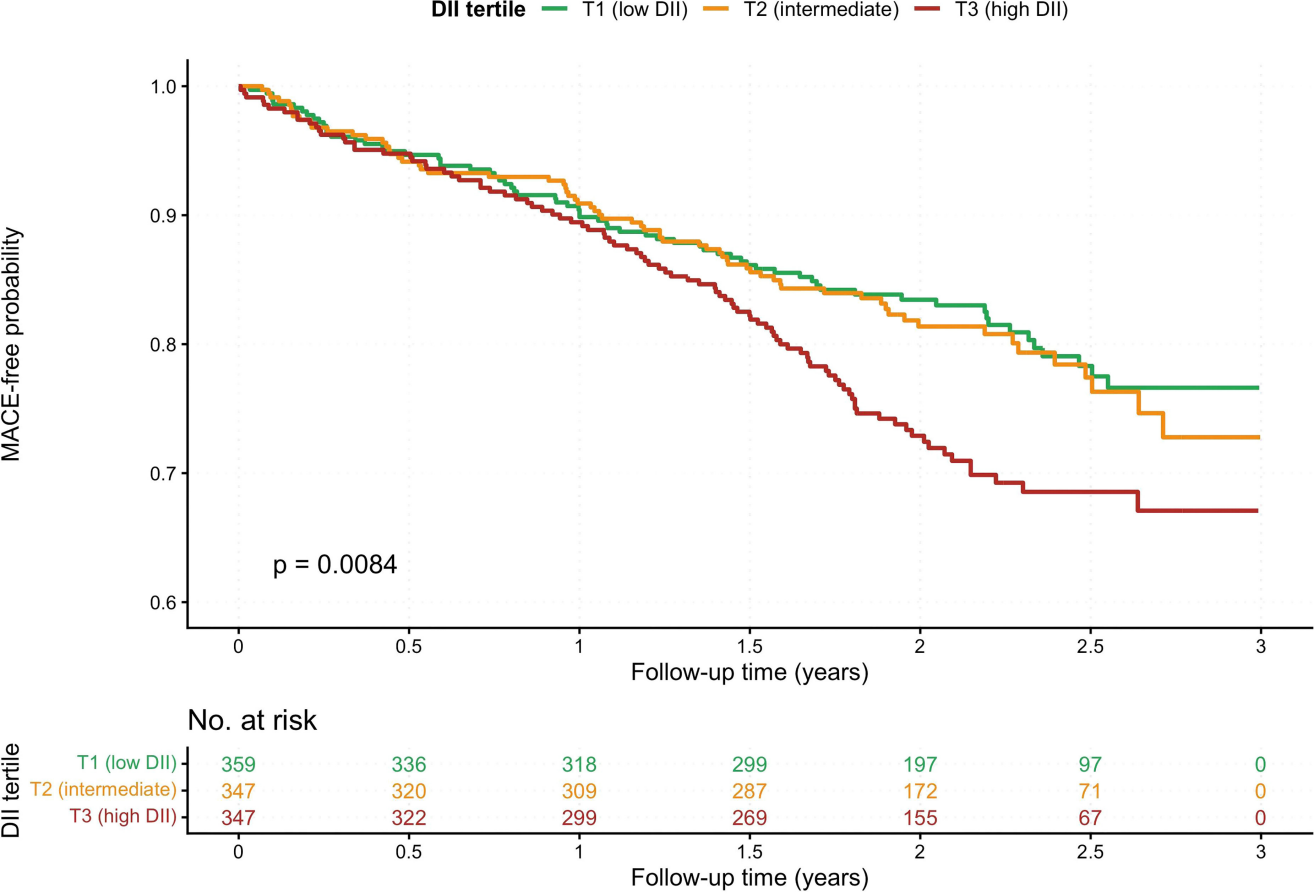
Kaplan–Meier curves for cumulative MACE-free survival, stratified by tertiles of the Dietary Inflammatory Index, in the Chinese PCI validation cohort (*n* = 1,053). The log-rank P value compares the three tertile groups. Numbers of patients at risk are shown beneath the figure. MACE = major adverse cardiovascular events (a composite of all-cause death, recurrent myocardial infarction, and repeat revascularization).

During a median follow-up of 23.9 months (IQR 19.0–29.5), 231 of 1,053 patients (21.9%) experienced MACE, including 76 (7.2%) all-cause deaths, 100 (9.5%) recurrent MI events, and 101 (9.6%) repeat revascularizations. Cox proportional-hazards models adjusted for age, sex, current smoking, diabetes mellitus, prior MI, prior stroke, eGFR, and SYNTAX score (Table 4) showed that each one-unit higher DII was independently associated with 19% higher hazards of MACE (HR 1.19, 95% CI 1.10–1.28), 24% higher hazards of recurrent MI (HR 1.24, 95% CI 1.10–1.39), 14% higher hazards of repeat revascularization (HR 1.14, 95% CI 1.02–1.28), and 19% higher hazards of all-cause mortality (HR 1.19, 95% CI 1.04–1.35). Each 10-unit higher LE8 score was associated with significantly lower hazards of MACE (HR 0.78, 95% CI 0.67–0.91), recurrent MI (HR 0.73, 95% CI 0.58–0.92), and repeat revascularization (HR 0.74, 95% CI 0.59–0.94), but the LE8–all-cause mortality association did not reach statistical significance (HR 0.84, 95% CI 0.65–1.09). Kaplan–Meier curves stratified by DII tertile showed progressively lower MACE-free survival with increasing DII (log-rank P = 0.0084; Figure 4), with separation emerging within the first year and widening throughout follow-up.

**TABLE 4:** Cox proportional-hazards models for the associations of the Dietary Inflammatory Index and Life’s Essential 8 score with adverse cardiovascular outcomes during follow-up in the Chinese PCI validation cohort (*n* = 1,053).

Endpoint	Events/N (%)	DII (per 1-unit) HR (95% CI), *P*	LE8 (per 10-unit) HR (95% CI), *P*
MACE	231 / 1,053 (21.9)	1.19 (1.10, 1.28), < 0.001	0.78 (0.67, 0.91), 0.001
All-cause mortality	76 / 1,053 (7.2)	1.19 (1.04, 1.35), 0.011	0.84 (0.65, 1.09), 0.192
Recurrent MI	100 / 1,053 (9.5)	1.24 (1.10, 1.39), < 0.001	0.73 (0.58, 0.92), 0.008
Repeat revascularization	101 / 1,053 (9.6)	1.14 (1.02, 1.28), 0.025	0.74 (0.59, 0.94), 0.012

Hazard ratios are expressed per 1-unit increase in DII and per 10-unit increase in LE8 score. All models were adjusted for age, sex, current smoking, diabetes mellitus, prior MI, prior stroke, eGFR, and SYNTAX score. The proportional-hazards assumption was verified by Schoenfeld residuals. CI, confidence interval; DII, Dietary Inflammatory Index; eGFR, estimated glomerular filtration rate; HR, hazard ratio; LE8, Life’s Essential 8; MACE, major adverse cardiovascular events (a composite of all-cause death, recurrent myocardial infarction, and repeat revascularization); MI, myocardial infarction; PCI, percutaneous coronary intervention; SYNTAX, Synergy between Percutaneous Coronary Intervention with Taxus and Cardiac Surgery.

## 4 Discussion

In two independent hypertensive cohorts [a U.S. nationally representative sample (NHANES, *n* = 12,376) and a Chinese single-center PCI cohort (*n* = 1,053)], a higher DII was linearly and independently associated with poorer CVH as captured by the AHA LE8 score. In the validation cohort, DII also independently predicted MACE, recurrent MI, repeat revascularization, and all-cause mortality during follow-up, extending the evidence from cross-sectional CVH to incident cardiovascular events. To our knowledge, this is the first study to externally validate the DII–CVH relationship in hypertension and to demonstrate that DII carries prognostic information beyond established clinical risk factors, including the SYNTAX score, in patients with established coronary disease.

Hypertension is a major contributor to global CVD (31) and promotes vascular stiffness and endothelial dysfunction that accelerate atherosclerosis (32). Inflammation is increasingly recognized as a central driver of hypertensive vascular injury (33), rendering anti-inflammatory pathways an attractive therapeutic target. The AHA LE8 framework (3) captures CVH more granularly than the earlier Life’s Simple 7 metric, and higher LE8 scores correlate with reduced cardiovascular risk (34, 35), protection against treatment-resistant hypertension (36), and lower cardiovascular and all-cause mortality in hypertensive populations (5, 37). The DII–CVH association was preserved across the two cohorts, which differ in age, sex distribution, ethnicity, and disease severity, supporting the robustness of the relationship in these settings.

Mediterranean and other anti-inflammatory dietary patterns improve metabolic health, lower platelet and leukocyte counts (9), and modulate the gut microbiota (38), offering plausible biological pathways linking dietary inflammatory potential to CVH. The DII shows positive associations with interleukin-6, tumor necrosis factor-α (39), WBC count, and neutrophil count (40), and pro-inflammatory dietary patterns have been linked to elevated cardiovascular risk in PREDIMED (41) and U.S. adult cohorts (42), to severe abdominal aortic calcification (43), and to atherosclerotic CVD specifically among hypertensive individuals (44). Mechanistically, accumulated inflammatory cytokines drive endothelial dysfunction and vulnerable-plaque formation (45), whereas anti-inflammatory dietary modification ameliorates oxidative stress, arterial stiffness, and endothelial dysfunction (46, 47).

Although the DII–CVH association was preserved in the PCI cohort, the point estimates were modestly attenuated (continuous OR 1.29 vs. 1.39 in NHANES; T3 vs. T1 OR 2.99 vs. 3.84). Such attenuation is expected when an exposure–outcome relationship established in a broad population is examined in a uniformly high-risk subset, in which the dynamic range of the exposure tends to be compressed and several LE8 components are shifted into suboptimal territory by underlying disease. The cross-cohort consistency in direction, gradient, and linearity makes a population-specific artifact unlikely as an explanation for the discovery findings.

Because diet quality (HEI-2015) is one of the eight components of LE8, the DII and LE8 overlap by construction, and part of the observed association is therefore expected on arithmetic grounds. Several observations nonetheless suggest the relationship is not driven by this overlap alone. DII and HEI-2015 are conceptually distinct, as HEI-2015 reflects adherence to dietary guidelines whereas the DII reflects the inflammatory potential of intake. Moreover, the gradient was not confined to the dietary domain: across increasing DII tertiles (Table 1), the non-dietary LE8 subscores for physical activity, nicotine exposure, and sleep also declined, while white-blood-cell and neutrophil counts rose, indicating that a higher-DII diet was accompanied by poorer status across several non-dietary domains. We acknowledge, however, that the most rigorous test of independence would re-estimate the association using a modified LE8 that excludes the dietary component; this re-scoring was not feasible in the present datasets and represents a priority for future work.

The smoking and diabetes interactions seen in NHANES were not reproduced in the PCI cohort. Plausible explanations include compression of smoking-exposure heterogeneity by post-PCI smoking-cessation counseling and the more intensive glycemic control that is typically applied after PCI in patients with diabetes; both of these processes may diminish the contrast between strata that drives interaction signals at the population level. Because these subgroup interactions were exploratory and did not replicate across cohorts, however, these explanations remain speculative.

Mediation indirect effects through neutrophil count, BMI, and WBC count, although directionally consistent with NHANES, did not reach statistical significance in the PCI cohort. This likely reflects the smaller sample size and partial saturation of inflammatory and metabolic mediators by guideline-directed pharmacotherapy [statins, angiotensin-converting enzyme (ACE) inhibitors/angiotensin receptor blockers (ARBs), antiplatelet agents], with consequent decoupling of the diet–inflammation–CVH pathway in established secondary prevention. The direct effect of DII on CVH remained large and statistically significant in every mediation model, consistent with a substantial component of the association acting independently of the candidate mediators tested.

An extension of this work is the demonstration that DII, captured by a single score, independently predicts MACE, recurrent MI, repeat revascularization, and all-cause mortality in hypertensive patients with established CAD, after adjustment for age, sex, smoking, diabetes, prior MI, prior stroke, eGFR, and the SYNTAX score, a widely used angiographic measure of coronary disease severity. Each one-unit increase in DII corresponded to 19–24% higher hazards across the four endpoints, and Kaplan–Meier curves showed separation among DII tertiles emerging within the first year of follow-up and widening thereafter. These observations extend the evidence from cross-sectional CVH to incident cardiovascular events, and support consideration of dietary inflammatory potential in secondary-prevention risk assessment.

LE8 itself was significantly associated with MACE, recurrent MI, and repeat revascularization, but its association with all-cause mortality did not reach statistical significance in the PCI cohort. This dissociation likely reflects a structural feature of LE8 in established secondary prevention: blood pressure, blood glucose, and blood lipids (three of the four LE8 health-factor components) are aggressively standardized by guideline-directed pharmacotherapy, narrowing their dynamic range and attenuating their discriminative power for the most distal endpoint, which is also influenced by competing non-cardiovascular causes. DII, by contrast, reflects a behavioral exposure largely unaffected by pharmacotherapy, and showed consistent associations with all four endpoints in our analysis. These observations suggest that DII may provide prognostic information beyond that captured by LE8 in this setting, although confirmation in larger and multi-center cohorts is needed.

The translation of these findings into clinical practice warrants caution. The DII is principally a research instrument: its calculation requires up to 28 food and nutrient parameters standardized against a global reference database, and it has no point-of-care implementation or validated cut-point that would define an actionable level of dietary inflammation for an individual patient. Our results therefore do not support routine measurement of the DII in practice. Rather, they identify the inflammatory quality of the diet as one plausible mechanism through which established patterns such as the Mediterranean and DASH diets confer cardiovascular benefit, and they reinforce existing advice to prioritize minimally processed, plant-predominant diets in hypertension. Translating these associations into practice will require simplified, food-based tools and prospective studies testing whether lowering the DII can improve cardiovascular health and outcomes.

Strengths of this study include the use of two large hypertensive cohorts, prespecified harmonization of variable definitions and analytic models across cohorts, and the extension to adverse cardiovascular endpoints in the validation cohort. Several limitations should be acknowledged. First, NHANES is cross-sectional and lacks longitudinal follow-up, precluding direct cross-cohort prognostic comparison. Second, the validation cohort is a single-center retrospective cohort with retrospective ascertainment of outcomes, which is susceptible to information bias and selection effects; multicenter prospective replication is required to confirm generalizability. Third, dietary intake in the validation cohort was captured by two consecutive 24-h recalls performed at the bedside during the index admission, covering the 48 h immediately preceding hospitalization. Although this approach has been adopted in similar cohorts of patients with acute coronary events, dietary intake during the 2 days before an acute event may not perfectly reflect long-term habitual diet, and the possibility of reverse causality (whereby prodromal symptoms altered intake) cannot be entirely excluded. The cross-cohort consistency of the DII–CVH and DII–outcome associations, however, makes it unlikely that this consideration substantially distorted our findings. Fourth, residual confounding from unmeasured factors, particularly lifestyle, socioeconomic, and environmental exposures, cannot be fully excluded. Fifth, because the DII, the candidate mediators, and LE8 were measured at the same cross-sectional time point in NHANES, the mediation analyses cannot establish temporal ordering or causal pathways and should be regarded as exploratory; the candidate mediators tested are also unlikely to capture the full mechanistic landscape linking diet to CVH and to outcomes. In addition, BMI is itself a component of the LE8 outcome and lies downstream of diet rather than serving as a purely mechanistic intermediate, so its mediation estimate should be interpreted with particular caution. Sixth, in both cohorts the DII reflects a single baseline assessment rather than a time-updated exposure; within-person day-to-day variation introduces random measurement error that is most likely non-differential with respect to outcome and would tend to bias the associations toward the null. Seventh, we did not include a non-inflammatory dietary comparator such as a Mediterranean or DASH score, so although our findings are consistent with an inflammation-related pathway, they cannot establish that the prognostic information carried by the DII is specific to inflammation rather than to overall diet quality; the inflammatory interpretation is therefore plausible but not established, and would be further clarified by direct comparison with such dietary-pattern scores.

## 5 Conclusion

In two independent hypertensive cohorts (a U.S. nationally representative sample and a Chinese secondary-prevention PCI cohort), a higher Dietary Inflammatory Index was independently and linearly associated with poorer cardiovascular health as measured by the AHA Life’s Essential 8 score. In the PCI cohort, DII also predicted MACE, recurrent myocardial infarction, repeat revascularization, and all-cause mortality, independently of established clinical risk factors and angiographic disease severity. As both cohorts are observational, these findings show association rather than causation and require prospective, ideally randomized, confirmation before any clinical recommendation.

## Data Availability

Publicly available datasets were analyzed in this study. This data can be found at: the U.S. Centers for Disease Control and Prevention at http://www.cdc.gov/nchs/nhanes/index.htm. De-identified data from the Chinese PCI cohort are available from the corresponding author on reasonable request, subject to applicable institutional and ethical approvals.
