# Supplementary material for: Dietary Inflammatory Index, cardiovascular health, and long-term outcomes in hypertension: external validation in a Chinese percutaneous coronary intervention cohort
Source: Front Nutr. 2026 Jul 28;13:1880961. doi: 10.3389/fnut.2026.1880961 (PMC13457097; doi:10.3389/fnut.2026.1880961)
Supplement: Supplementary file 1 [file Table_1.docx]

Supplementary Material

# 1 Supplementary Figures and Tables

## 1.1 Supplementary Figures

**Supplementary Figure 1.** Forest plot of subgroup analyses of the association between the Dietary Inflammatory Index (per 1-unit increase) and suboptimal cardiovascular health (LE8 <80) in the NHANES discovery cohort. Each model was adjusted for age, sex, race, education, body-mass index, smoking status, poverty income ratio, diabetes, coronary artery disease, and stroke (with the stratifying variable excluded from the covariate set), and incorporated NHANES sampling weights. P for interaction was assessed by entering a multiplicative DII × subgroup interaction term in the fully adjusted model. Significant interactions were observed for smoking and diabetes (Pinteraction <0.05).

## 1.2 Supplementary Tables

**Supplemental Table 1.** Sensitivity analysis: weighted multivariable logistic regression of the Dietary Inflammatory Index against suboptimal cardiovascular health redefined as LE8 <50, in the NHANES discovery cohort (n = 12,376).

| Exposure | Model 1, OR (95% CI), P | Model 2, OR (95% CI), P | Model 3, OR (95% CI), P |
| --- | --- | --- | --- |
| DII (per 1-unit) | 1.31 (1.26, 1.37), <0.001 | 1.28 (1.22, 1.34), <0.001 | 1.29 (1.23, 1.35), <0.001 |
| DII tertiles |  |  |  |
| T1 (lowest) | 1.00 (Reference) | 1.00 (Reference) | 1.00 (Reference) |
| T2 | 1.86 (1.53, 2.26), <0.001 | 1.72 (1.38, 2.15), <0.001 | 1.73 (1.39, 2.16), <0.001 |
| T3 (highest) | 3.03 (2.53, 3.63), <0.001 | 2.77 (2.26, 3.40), <0.001 | 2.82 (2.29, 3.48), <0.001 |
| P for trend | <0.001 | <0.001 | <0.001 |

Model 1: unadjusted. Model 2: age, sex, race, education, BMI, smoking status, and PIR. Model 3: Model 2 covariates plus diabetes, CAD, and stroke. NHANES analyses incorporated complex survey-design weights.

BMI, body-mass index; CAD, coronary artery disease; CI, confidence interval; CVH, cardiovascular health; DII, Dietary Inflammatory Index; LE8, Life’s Essential 8; OR, odds ratio; PIR, poverty income ratio.

**Supplemental Table 2.** Mediation analysis examining the role of inflammatory and metabolic markers in the association between the Dietary Inflammatory Index and suboptimal cardiovascular health (LE8 <80) in the NHANES discovery cohort (n = 12,376; weighted).

| Mediator | Indirect effect (95% CI) | Direct effect (95% CI) | Total effect (95% CI) | Proportion mediated, P |
| --- | --- | --- | --- | --- |
| WBC | 0.000201 (−0.000488, 0.001) | 0.022705 (0.019765, 0.026) | 0.022907 (0.019985, 0.026) | 0.81%, 0.580 |
| Neutrophil | 0.000472 (0.000246, 0.001) | 0.022486 (0.019914, 0.025) | 0.022958 (0.020366, 0.026) | 2.02%, <0.001 |
| BMI | 0.00180 (0.00135, 0.0023) | 0.02112 (0.01860, 0.0237) | 0.02291 (0.02030, 0.0256) | 7.83%, <0.001 |

Mediation effects were estimated using weighted multivariable models with the R package ‘mediation’, 500 bootstrap simulations, and percentile-based 95% confidence intervals.

BMI, body-mass index; CI, confidence interval; CVH, cardiovascular health; DII, Dietary Inflammatory Index; LE8, Life’s Essential 8; WBC, white blood cell.

**Supplemental Table 3.** Weighted multivariable linear regression of the Dietary Inflammatory Index as a predictor of candidate mediators in the NHANES discovery cohort (n = 12,376).

| Outcome (mediator) | Model 1, β (95% CI), P | Model 2, β (95% CI), P | Model 3, β (95% CI), P |
| --- | --- | --- | --- |
| WBC, ×10⁹/L | 0.07 (0.04, 0.11), <0.001 | 0.06 (0.03, 0.10), <0.001 | 0.06 (0.03, 0.10), <0.001 |
| Neutrophil, ×10⁹/L | 0.06 (0.03, 0.08), <0.001 | 0.05 (0.03, 0.07), <0.001 | 0.05 (0.03, 0.07), <0.001 |
| BMI, kg/m² | 0.40 (0.30, 0.50), <0.001 | 0.32 (0.23, 0.42), <0.001 | 0.32 (0.23, 0.41), <0.001 |

Coefficients (β) represent the change in the mediator per 1-unit increase in DII. Model 1: unadjusted. Model 2: age, sex, race, education, smoking status, and PIR (BMI omitted when modelled as outcome). Model 3: Model 2 covariates plus diabetes, CAD, and stroke. NHANES analyses incorporated complex survey-design weights.

BMI, body-mass index; CAD, coronary artery disease; CI, confidence interval; DII, Dietary Inflammatory Index; PIR, poverty income ratio; WBC, white blood cell.

**Supplemental Table 4.** Weighted multivariable logistic regression of candidate mediators as predictors of suboptimal cardiovascular health (LE8 <80) in the NHANES discovery cohort (n = 12,376).

| Mediator (per 1-unit increase) | Model 1, OR (95% CI), P | Model 2, OR (95% CI), P | Model 3, OR (95% CI), P |
| --- | --- | --- | --- |
| WBC, ×10⁹/L | 1.10 (1.06, 1.14), <0.001 | 1.09 (1.05, 1.13), <0.001 | 1.08 (1.04, 1.12), <0.001 |
| Neutrophil, ×10⁹/L | 1.16 (1.10, 1.21), <0.001 | 1.13 (1.08, 1.19), <0.001 | 1.12 (1.07, 1.18), <0.001 |
| BMI, kg/m² | 1.10 (1.09, 1.12), <0.001 | 1.10 (1.08, 1.11), <0.001 | 1.09 (1.08, 1.11), <0.001 |

Odds ratios (OR) represent the change in odds of suboptimal CVH (LE8 <80) per 1-unit increase in the mediator. Model 1: unadjusted. Model 2: age, sex, race, education, smoking status, and PIR. Model 3: Model 2 covariates plus diabetes, CAD, and stroke. NHANES analyses incorporated complex survey-design weights.

BMI, body-mass index; CAD, coronary artery disease; CI, confidence interval; CVH, cardiovascular health; LE8, Life’s Essential 8; OR, odds ratio; PIR, poverty income ratio; WBC, white blood cell.

**Supplemental Table 5. Subgroup analyses of the association between the Dietary Inflammatory Index (per 1-unit increase) and suboptimal cardiovascular health (LE8 <60), stratified by clinical and demographic characteristics, in the Chinese PCI validation cohort.**

| Subgroup | Stratum | N | Events | OR (95% CI), P | P-interaction |
| --- | --- | --- | --- | --- | --- |
| Sex | Male | 836 | 483 | 1.29 (1.17, 1.41), <0.001 | 0.647 |
|  | Female | 217 | 149 | 1.35 (1.13, 1.60), <0.001 |  |
| Age | <65 years | 584 | 336 | 1.32 (1.18, 1.47), <0.001 | 0.726 |
|  | ≥65 years | 469 | 296 | 1.26 (1.12, 1.42), <0.001 |  |
| Diabetes | Non-diabetic | 602 | 307 | 1.24 (1.12, 1.37), <0.001 | 0.261 |
|  | Diabetic | 451 | 325 | 1.37 (1.19, 1.57), <0.001 |  |
| Smoking | Non-smoker | 578 | 339 | 1.27 (1.14, 1.41), <0.001 | 0.555 |
|  | Current smoker | 475 | 293 | 1.35 (1.19, 1.53), <0.001 |  |
| BMI | <25 kg/m² | 404 | 168 | 1.26 (1.11, 1.43), <0.001 | 0.588 |
|  | ≥25 kg/m² | 560 | 378 | 1.38 (1.22, 1.56), <0.001 |  |
| Admission diagnosis | STEMI | 11 | 8 | Insufficient events | 0.442 |
|  | NSTEMI / unstable angina | 1,042 | 624 | 1.28 (1.19, 1.39), <0.001 |  |
| Prior MI | No prior MI | 813 | 478 | 1.30 (1.18, 1.42), <0.001 | 0.481 |
|  | Prior MI | 240 | 154 | 1.24 (1.05, 1.47), 0.012 |  |

Each model was adjusted for age, sex, current smoking, diabetes mellitus, prior MI, prior stroke, and eGFR (with the stratifying variable excluded from the covariate set). P for interaction was assessed by entering a multiplicative DII × subgroup interaction term in the fully adjusted model.

BMI, body-mass index; CI, confidence interval; eGFR, estimated glomerular filtration rate; MI, myocardial infarction; NSTEMI, non-ST-elevation myocardial infarction; OR, odds ratio; STEMI, ST-elevation myocardial infarction.

**Supplemental Table 6.** Mediation analysis examining the role of inflammatory and metabolic markers in the association between the Dietary Inflammatory Index and suboptimal cardiovascular health (LE8 <60) in the Chinese PCI validation cohort.

| Mediator | N | Indirect effect (95% CI), P | Direct effect (95% CI), P | Total effect (95% CI), P | Proportion mediated, % (95% CI), P |
| --- | --- | --- | --- | --- | --- |
| Neutrophil count, ×10⁹/L | 995 | 0.0018 (−0.0018, 0.0056), 0.320 | 0.0521 (0.0347, 0.0697), <0.001 | 0.0539 (0.0362, 0.0702), <0.001 | 3.30 (−3.32, 11.47), 0.320 |
| BMI, kg/m² | 964 | 0.0022 (−0.0024, 0.0069), 0.384 | 0.0510 (0.0334, 0.0681), <0.001 | 0.0531 (0.0344, 0.0714), <0.001 | 4.05 (−5.44, 12.26), 0.384 |
| WBC count, ×10⁹/L | 995 | 0.0015 (−0.0018, 0.0053), 0.372 | 0.0524 (0.0348, 0.0703), <0.001 | 0.0539 (0.0362, 0.0701), <0.001 | 2.80 (−3.41, 10.51), 0.372 |

Mediation models were adjusted for age, sex, current smoking, and diabetes mellitus. Indirect effects were estimated by the non-parametric bootstrap (500 simulations, percentile-based 95% confidence intervals).

BMI, body-mass index; CI, confidence interval; WBC, white blood cell.
